# A school-based intervention for a better future: study protocol of Sintra Grows Healthy

**DOI:** 10.1186/s12889-020-09715-0

**Published:** 2020-10-27

**Authors:** Raquel J. Ferreira, Telma Nogueira, Vitória Dias da Silva, Mariana Liñan Pinto, Joana Sousa, Ana Margarida Pereira, Paulo Jorge Nogueira, Rute Borrego, Ana Raposo, João Martins, Marcos Onofre, Adilson Marques, António Rodrigues, Ana Quitério, António Pereira

**Affiliations:** 1 Câmara Municipal de Sintra, Departamento de Educação, Juventude e Desporto, Largo Dr. Virgílio Horta, 2714-501 Sintra, Portugal; 2 Escola Superior de Tecnologia da Saúde de Lisboa, Instituto Politécnico de Lisboa, Avenida D. João II, Lote 4.69.01, 1990-096 Lisbon, Portugal; 3 Laboratório de Nutrição, Faculdade de Medicina, Universidade de Lisboa, Avenida Professor Egas Moniz, Edifício Egas Moniz, ala C, piso 2, 1649-028 Lisbon, Portugal; 4 Instituto Saúde Ambiental, Faculdade de Medicina, Universidade de Lisboa, Avenida Professor Egas Moniz, Edifício Egas Moniz, ala C, piso 0, 1649-028 Lisbon, Portugal; 5 Laboratório de Biomatemática Faculdade de Medicina, Universidade de Lisboa, Avenida Professor Egas Moniz, Edifício Egas Moniz, ala C, piso 0, 1649-028 Lisbon, Portugal; 6 Instituto de Medicina Preventiva e Saúde Pública, Faculdade de Medicina, Universidade de Lisboa, Avenida Professor Egas Moniz, Edifício Egas Moniz, ala C, piso 0, 1649-028 Lisbon, Portugal; 7 Escola Superior de Comunicação Social, Instituto Politécnico de Lisboa, Campus de Benfica do IPL, 1549-014 Lisbon, Portugal; 8 Faculdade de Motricidade Humana, Universidade de Lisboa, Estrada da Costa, 1499-002 Cruz Quebrada, Dafundo Portugal; 9 Laboratório de Pedagogia, Faculdade de Motricidade Humana & UIDEF, Instituto de Educação, Universidade de Lisboa (FMH), Estrada da Costa, 1499-002 Cruz Quebrada, Dafundo Portugal; 10 CIPER, Faculdade de Motricidade Humana, Universidade de Lisboa, Estrada da Costa, 1499-002 Cruz Quebrada, Dafundo Portugal; 11 Agrupamento de Centros de Saúde de Sintra, Estrada de Mem Martins 247 - 6° andar, 2725-391 Mem Martins, Portugal

**Keywords:** Childhood obesity, Health promotion, Participatory research, Local government, Physical activity, Nutrition, Lifestyle, Behaviour

## Abstract

**Background:**

Preventing childhood obesity is a public health challenge of the twenty-first century and it must be a priority. Governments play a major role in creating and supporting a healthy school environment and should prioritise actions to improve children’s health. Sintra Grows Healthy aims to promote healthy lifestyles to prevent childhood obesity and improve children’s health-related quality of life and social and emotional skills, through the development of a school evidence-based and sustainable model.

**Methods:**

This protocol describes a quasi-experimental design and community-based participatory research. The participants included in the study are the school community of Portuguese public primary schools from the municipality of Sintra. Data will be collected on demographic and socio-economic characterization, nutritional status, eating habits and behaviours, physical activity, sedentary behaviours and sleep, health-related quality of life, and social and emotional skills.

**Discussion:**

There is evidence to support interventions in school settings as strategies for obesity prevention. Up-to-date homogeneous and community-based interventions for preventing childhood obesity are lacking, therefore Sintra Grows Healthy intends to fill this gap. Furthermore, Sintra Grows Healthy aims to contribute with relevant scientific findings that will allow the development of better strategies for policymakers and society to manage this major public health problem.

## Background

Childhood obesity is one of the most serious global public health challenges of the twenty-first century. In just 40 years the number of schoolchildren and adolescents with obesity has increased more than ten-fold [1]. It is recognised that obesity is a complex system of distinct and interconnecting factors including food consumption and physical activity patterns, genetic factors, environmental structures, cultural values, social inequalities, social class differences, economic factors, social gradients, and stress [2]. Current literature indicates that obesity prevalence is higher amongst adolescent boys [3], particularly those from ethnic minorities [4]. Ethnicity is associated with differences in food habits and behaviours and cultural influences may contribute to higher obesity prevalence among children from minority populations [5]. Also, evidence suggests that children from lower socioeconomic status tend to have poorer dietary habits and lower levels of physical activity [6].

Childhood obesity increases the likelihood of obesity in adulthood and is strongly associated with the development of non-communicable diseases such as cardiovascular disease [7] and diabetes [8]. Preventing obesity has direct benefits for children’s health and wellbeing in childhood and subsequently in adulthood [9]. Also, as reported by the Organisation for Economic Co-operation and Development (OECD), preventing obesity is extremely beneficial for the economic system [10]. In Portugal, the prevalence of obesity and pre-obesity in children under 10 years old is, respectively, 7.7 and 17.3% [11]; 12% of children aged between 6 and 8 years are obese and 29.6% are overweight [12]. In the primary schools of Sintra’s municipality, the prevalence estimates change to 12.6% obese and 23% pre-obesity [13].

The United Nations Convention on the Rights of the Child establishes the children’s rights of protection, education, health, and health care, shelter, and adequate nutrition [14]. Rome Declaration on Nutrition reinforces that collaboration between governments, the private sector, civil society, and communities are crucial for improving nutrition [15]. Therefore, governments play a major role in creating and supporting a healthy environment and should prioritise actions to improve children’s healthy preference learning and to ensure adequate availability of healthy food [16]. Regarding schools, governments should set food standards, and provide healthy eating education, influencing a cultural shift towards healthier food preferences [16]. International guidelines state that municipalities are promising spaces for improving the nutritional status of children and that they must assume a leading role in fighting childhood obesity [17]. In Portugal, the city council is responsible for “support social, cultural, educational, sporting, recreational or other activities of interest to the municipality, including those that contribute to health promotion and disease prevention” [18] including “to participate in programmes to promote public health, community health, and healthy living habits” [19]. Governments can take various actions to create a healthy school environment namely setting physical activity standards [20, 21] and nutritional standards for school meals and minimising the exposure to advertisements of foods and beverages high in fat, sugar and salt [9].

Childhood is one of three sensitive periods of the life-course identified as critical points of intervention that can influence obesity risk in the context of the encompassing obesogenic environment [22]. Schools constitute an important setting for promoting physical, social, cognitive, and mental health and establishing healthy behaviour [23], protect and support good nutrition in children and their families and communities [24, 25]. Tackling nutrition issues in childhood requires coherent action in the school setting, namely through the development of food and nutrition policies [24]. Schools are also considered to be an important context to promote active lifestyles [20, 21]. Regular practice of physical activity can have multiple benefits [26] but most children fail to meet the recommended 60 min of moderate-to-vigorous physical activity daily [27, 28] and present low levels of basic motor competences [29].

Health promotion must have five essential interrelated actions as established by the Ottawa Charter for Health Promotion: building healthy public policy, creating supportive environments, strengthening community action, developing personal skills and reorienting health services [30]. Those concepts must also be applied in nutrition education [31] and health-promoting schools [32]. The health-promoting school (HPS) model has been a notable international health initiative [33]. There is evidence to sustain that this model is effective in improving certain areas of health, namely reducing children’s BMI and increasing children’s physical activity [34–36]. An HPS is a school that implements a structured and systematic plan for the health, well-being, and the development of social capital of all pupils and teaching and non-teaching staff [37]. HPS also creates an environment that encourages personal and social development, leading to better health [38].

Comprehensive school-based nutrition programmes involve the school community and address multiple components (curriculum, quality, and quantity of meals served and other actions addressing the school environment, such as vending machines and areas to practise physical activity) [25]. In what concerns the promotion of physical activity, a comprehensive multi-component approach that includes physical education, physical activity during school (e.g., recess), physical activity before and after school (e.g., active travel, school sports intramurals), staff involvement, and family and community engagement has also been recommended and implemented [35]. Interventions at school settings are strategies for obesity prevention [36]. Although childhood obesity may offer many opportunities to intervene, the heterogeneity present in some interventions, set in different contexts across the globe, does not account for a global mechanism for stakeholders to implement [22]. Nevertheless, multifaceted interventions that combine diet and physical activity are particularly associated with a range of positive outcomes, including healthier weight, diet, and levels of physical activity among schoolchildren [9, 36]. Also, diet combined with physical activity interventions may be effective to reduce the risk of obesity in children aged 6 to 12 years [36]. Evidence shows that interventions to improve child nutrition and physical activity are more effective if they involve the whole community (students, teachers, school staff, and parents) [22]. Moreover, it is known that multi-component strategies [36, 37], implemented in collaboration with school community elements over sustained periods [39, 40], and initiated in early age [22] are likely to have the greatest impact. The intensity and duration of the intervention are characteristics that must necessarily be considered when planning it. Extended programmes with frequent interventions seem to be more successful than shorter durations or with time-spaced activities [39, 40]. Additionally, social and emotional skills have established links to mental health and health-related behaviour, affecting physical health [41]. Today’s children will need a balanced set of cognitive, social, and emotional skills to succeed in modern life and to meet the challenges of the twenty-first century [42].

Up-to-date homogenous prevention strategies for childhood overweight and obesity are lacking and more community-environment-based preventive approaches are necessary [43, 44]. Also, future research should focus on intervention sustainability [45] and impact evaluation [46]. In Portugal, impact evaluation of interventions is missing, therefore, it is crucial to provide their effectiveness in behaviour change [46]. Thus, a school-based intervention named Sintra Grows Healthy (SGH) was developed to contribute to a community-environment-based preventive approach. SGH will develop and evaluate the effectiveness of an evidence-based and sustainable school intervention model that could be reproducible in similar contexts. SGH aims to improve children’s health-related quality of life by promoting key dietary and physical activity behaviours and simultaneously enhancing social and emotional skills, preventing the increase of childhood obesity prevalence. Considering that the third United Nations Sustainable Development Goal (good health and well-being) involves a broad range of social determinants covered by the remaining goals [47], SGH also intends to contribute as an integrated response to the 2030 Agenda for Sustainable Development [48].

Therefore, this study protocol aimed to describe the design and methodology of the Sintra Grows Healthy.

## Methods

### Design

This protocol reports a community intervention with a quasi-experimental design and community-based participatory research. The study was developed by the municipality of Sintra (the project owner) with a partnership between health and academic entities as stakeholders: Sintra Health Centres Group, College of Communication and Media Studies (Polytechnic Institute of Lisbon), Lisbon School of Health Technology (Polytechnic Institute of Lisbon), Nutrition Laboratory of Faculty of Medicine (University of Lisbon), Faculty of Human Kinetics (University of Lisbon). Furthermore, SGH has the institutional support of organisations such as Ministry of Health, Portuguese National Programme for the Promotion of Healthy Eating, Portuguese National Programme for the Promotion of Physical Activity, Ministry of Education, Portuguese Council of Nutritionists, Portuguese Order of Psychologists, Food and Agriculture Organisation of the United Nations (FAO) – Portugal, NOVA National School of Public Health (NOVA University Lisbon) and Food, Farming & Forestry College (F3) (University of Lisbon).

### Participants

Sintra is the second Portuguese municipality with the largest number of inhabitants, considering children and young people [49] (62.834 under 15 years old in 2018 [50]), and is characterised by a multicultural population representing 8.7% of the total population of the municipality, more than double the percentage registered in Portugal [49]. Sintra’s School Network includes 20 public school clusters with a total of 96 kindergartens and primary schools. The study includes the respective subgroup of 83 primary schools from the municipality of Sintra (approximately 13,100 children). Firstly, we are recruiting 16 schools and our goal is to reach within 5 years the entire population - the 83 primary schools from the municipality of Sintra. The sample is selected by the schools’ convenience and split into an intervention group and a control group, guaranteeing the sample size required to reach the minimum difference of proportions between groups of 3–5% with at least 80% statistical power. Any children who meet the inclusion criteria (attend the primary schools belonging to the project and give written freely given, enlightened, informed consent for participation with children’s legal guardian approval and signature) and do not meet the exclusion criteria (to have a motor and/or intellectual disabilities and/or not present a valid consent for participation) are eligible (census procedure). As mentioned in the informed consent this study does not represent any risk, cost, or harm to the participants.

### Procedure

SGH aims to develop a sustainable model of intervention to promote healthy lifestyles in a school environment and to evaluate its impact. To achieve it, SGH community-based intervention focuses on community actors and prioritises three axes: 1) food and nutrition curricula named Health at the Table, 2) school food environment and 3) physical activity. Throughout the process, a specialised team will design a set of communication tools to inform, engage and motivate all the stakeholders involved in the process, from the institutional partners, to the local community, to children, families, teachers and other actors who play an important role in the school environment. The intervention follow-up will occur during each school year from the first to fourth grade. Also, SGH researchers will assure continuous monitoring and disclosure of a quarterly report for all intervention axes to the schools, to communicate and verify the school community engagement.

### Axis 1 – health at the table

This axis is focused on providing children a nutrition curriculum by integrating weekly sessions of food and nutrition education. To achieve a sustainable model those sessions will be developed by the responsible teacher of each class. Also, Health at the Table is a response to a mandatory curriculum component established in Portuguese law, called “the complementary offer” [51]. Complementary offer is considered a new mandatory attendance subject (not covered in the basic curricular matrices) that has its own identity and curriculum documents [51]. Through curricular flexibility and articulation, the complementary offer allows the curriculum to be enriched with the knowledge, skills and attitudes that contribute to achieving the competencies provided in the Profile of Students after Leaving Compulsory Education [52]. To ensure that nutrition education content is properly given to children, SGH developed a 50-h training that all teachers need to attend to be able to lecture those sessions. The training contents are based on scientific evidence related to the topics covered namely i) Food and culture, ii) Food, nutrition, and health, iii) Food and emotions, iv) Food cycle: from the producer to the consumer, v) Safe cooking and vi) Food sustainability. It also includes practical exercises, such as choosing a snack, consulting nutrition labelling, checking the adequacy to children’s energy and nutritional needs or presenting solutions to reduce food waste at school. SGH has also developed a reference manual that provides the contents organised for each schooling level and which also allows connection with the basic curricula subjects (Maths, Science and Portuguese). This manual comprises those six main areas, previously identified by the Portuguese Health Education Reference [53].

This axis will be monitored through a weekly submission in an SGH platform for each food and nutrition education sessions applied by the teachers. In this submission, teachers will fill in a form regarding the application of each session and will answer several questions, (e.g. date of application, improvement opportunities, children’s competencies achieved and activity adequacy).

### Axis 2 – school food environment

The municipality of Sintra has already developed several policies to provide children with a healthier school food environment such as assuring the quality of school meals, being all menus established by registered nutritionists/dietitians. In primary schools, a salt control policy has been implemented at school lunches. Moreover, Sintra assures professional ongoing training of cooks, family dinners at schools, and monthly healthy culinary workshops for families.

To continue that work, SGH will implement and monitor a healthy snacks policy. The construction of this policy is developed in a community-based participatory approach, co-designed, and includes different moments explained below.

- Snacks Diagnosis – Qualitative and quantitative evaluation of foods consumed by children in both morning and afternoon snacks.
- Snacks Assembly – Within the scope of health at the table, children will organise an assembly to discuss the quality of theirs snacks: what is a healthy snack, which causes lead to the consumption of unhealthy snacks by the class, and what they could implement to improve their snacks. At the end, children build a document, signed by the entire class, with the main conclusions of their debate to be discussed in an assembly with the school community.
- School Community Assembly – SGH research team will present the snacks diagnosis. Based on the document prepared by the children at the snacks assembly and on Directorate-General for Education guidance for school buffets [54], the school community will afterwards construct a healthy snacks policy. After construction and approval of the healthy snacks policy, it will be incorporated into the school regulation, assuring SGH sustainability.
- Healthy Snacks Policy Monitoring – To monitor policy compliance a self-regulation map based on the nutritional quality of snacks will be completed by the children daily. Children, supervised by their teachers, will categorise their snacks in a colour system: green (foods to be promoted), yellow (foods to limit) or red (foods not to be available at school) as established by the Directorate-General for Education guidance for school buffets [54].

### Axis 3 – physical activity

Physical education is a unique context where all children can have access to quality physical activity experiences guided by a teacher. Physical education has been shown to provide an effective means to promote physical activity inside and outside of school [55]. However, in Portugal, despite being compulsory, physical education often lacks implementation in many primary schools (grades 1 to 4). Some of the main reasons are related to the low perceived status of the subject compared to Language and Mathematics, lack of time and resources, general teacher’s perceived lack of competence to teach physical education, and, lack of continuous and contextualised professional development opportunities [56, 57]. The Directorate-General of Education [51] specified that physical education (and arts education) in primary school should be taught up to 5 h a week and that the collaboration of general teachers with specialist teachers in this field is a possibility. Thus, in this axis, the priority of the intervention was given to provide at least one weekly physical education session (45–60 min) - developed, implemented, and evaluated by the collaborative participation of general and specialist teachers – to children. The contents for physical education classes are the basic motor skills, such as running, jumping, rolling, kicking, grabbing and throwing, gymnastic exercises, and pre-sports games. To ensure SGH sustainability, a previously training (25 h) aimed at general and specialist teachers in the scope of the development of children’s basic motor competences and physical activity was conducted. Children’s basic motor competences (objectively measured) and physical activity levels in different contexts (reported by legal guardian) were measured in the beginning and at the end of the year (pre and post-intervention). Table 1 summarises the information regarding research questions, intervention axes, and expected results and outcomes.

**Table 1:** Research questions, intervention axes, and expected results and outcomes

**Research Questions**	Does the Sintra Grows Healthy model prevent the increase of childhood obesity prevalence?		
	Does the Sintra Grows Healthy model improve children’s social and emotional skills?		
	Does the Sintra Grows Healthy model improve children’s health-related quality of life?		
**Intervention Axes**	**Curricula Health at the Table**	**School Food Environment**	**Physical Activity**
	Weekly sessions of food education given by the teacher in a curricular context as a complementary offer.	Implementation and monitoring of a healthy snacks policy.	Weekly sessions of physical education in collaboration between general teacher and specialist teacher.
**Expected Results**	To develop and measure the impact of a school evidence-based and sustainable model to promote healthy lifestyles that could be reproducible in similar contexts.		
**Outcomes**	Prevent the increase of childhood obesity prevalence and improve children’s social and emotional skills and health-related quality of life.		

**Fig. 1:**
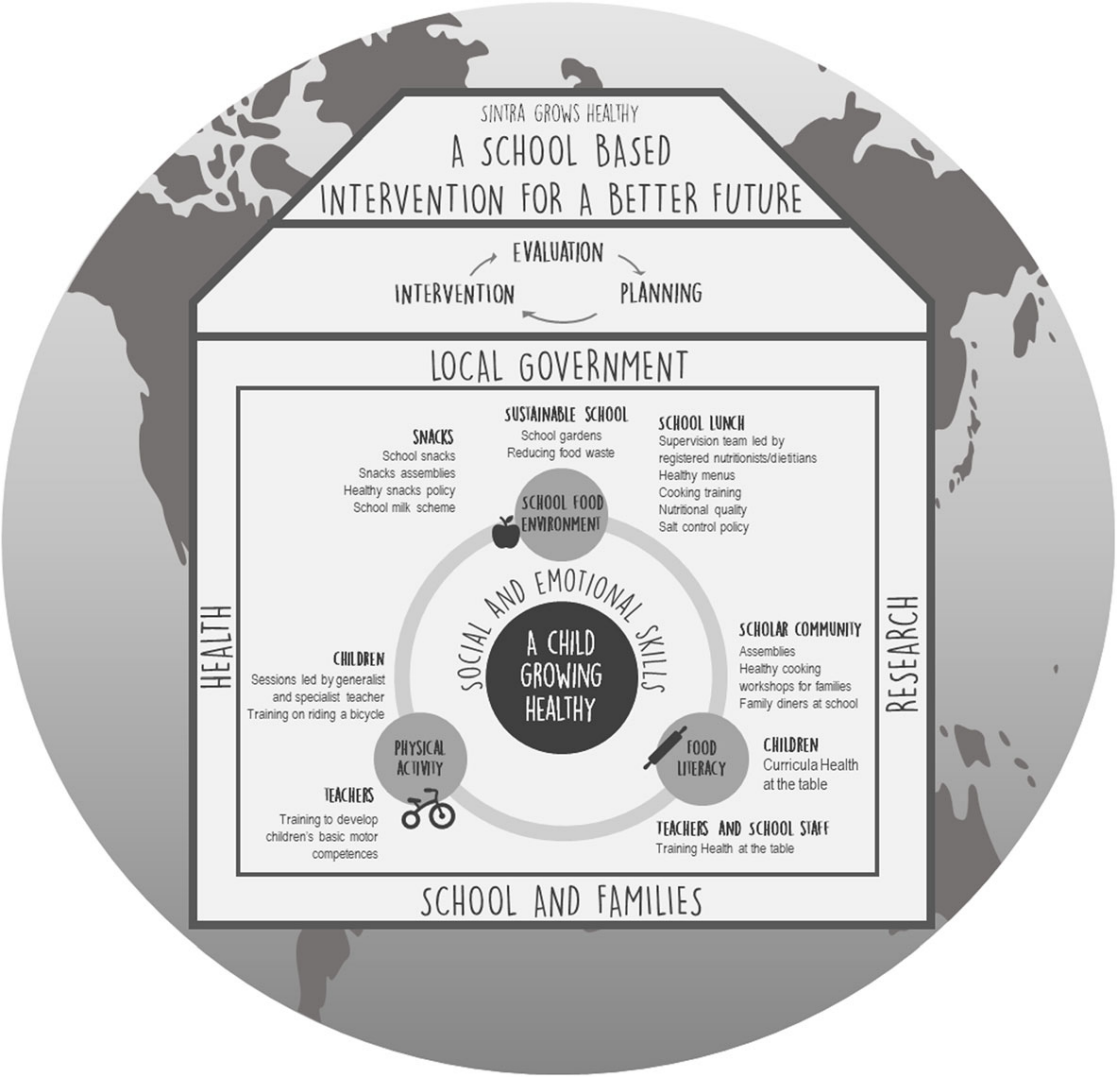
Sintra Grows Healthy framework of action

Figure 1 presents the SGH framework of action. This reinforces the importance of community-based participatory research and the crucial role of whole-of-government and whole-of-society to succeed in all intervention axes and achieve the expected results and outcomes.

### Data collection

Data collection will be based on structured questionnaires applied to children, children’s legal guardians, teachers, and school staff. The questionnaires evaluate the following dimensions: 1) sociodemographic and health-related data, 2) eating habits and behaviours, 3) movement behaviours: physical activity, sedentary behaviour and sleep 4) health-related quality of life, 5) social and emotional skills and 6) process evaluation. Children’s 7) anthropometric measurements, 8) snacks evaluation, and 9) basic motor competences will be collected by trained researchers to avoid deviations. To ensure that procedures are carried out strictly, teachers will receive specific training regarding the snack’s evaluation.

1. Sociodemographic and Health-Related Data

Child’s data (sex, date of birth, schooling level, previous chronic illnesses or health conditions), number of individuals in the household and children’s legal guardians data (age, nationality, professional status, education, professional activity, weight, height, family income) will be collected.

2) Eating Habits and Behaviours

An instrument will be applied to evaluate the adequacy of Mediterranean dietary patterns in children, reported by children’s legal guardian: KIDMED, a 16-item instrument with an index that ranges from zero to twelve Questions denoting a negative connotation concerning the Mediterranean diet are assigned a value of minusone, and those with a positive aspect plusone. The classification is divided into three levels: ≥8 optimal Mediterranean diet; four to seven, improvement needed to adjust intake to Mediterranean patterns; ≤three very low diet quality [58]. Also, a validated instrument for rapidly assessing the adherence to the Mediterranean dietary patterns in adulthood will be applied to the legal guardian. It is a 14-item scale and the answer to each of the items is scored with one in the case of fulfilling the criteria defined as a characteristic of this pattern or zero if it does not meet. Classification ≥ten is considered to be good adherence [59].

Children food preferences will be collected based on 40 common foods consumed in Portugal, using a 4-item scale (“likes very much”, “neither likes nor dislikes”, “does not like”, “I do not know”) applied to the legal guardian. Children’s involvement in family food choices and food preparation (e.g. how often they helped make family meal choices, how often they helped make family food purchases choices, how often they helped prepare or cook food at home) [60] and basic food skills proposed by age (e.g. measure ingredients, prepare sandwiches and simple snacks, read and follow recipes) [24] will be collected and treated as proxies for children food skills, reported by the legal guardian.

Parents’ food skills will be collected, namely, by meal planning habits, meal preparation practices (e.g. “Are you capable of cooking with leftovers?”), mechanical skills (e.g. cutting, peeling, baking from a recipe), ranked by the respondent on a scale ranging from “very limited” to “very good” [61, 62]. Besides, cooking attitudes will be also collected using a 5-item Likert scale (e.g. “I do not like cooking”, “I pretend to teach my child how to cook”, “My child does not need to know how to cook”).

3) Movement Behaviours: Physical Activity, Sedentary Behaviour, and Sleep

Total physical activity will be assessed by the question “In the last seven days, how many days did your child practised physical activity for at least 60 minutes?”. This is a valid and reliable question assessing physical activity [63] and it has been used in several international and national studies [28, 64]. Participation in organised physical activity (e.g. sports activities guided by a teacher/trainer outside of school), unorganized physical activity (e.g. non-guided activities outside of school) and school sports (e.g. activities systematically conducted in the context of school sport context) will also be collected by the questions “How many times a week does your child take part in… (1) organised physical activity? (2) unorganised physical activity? (3) school sports?”. Active travel to school will be assessed by the question: “How do you usually, travel from home to school?”. The response options will be walking, cycling, by public transportation, by car, and by motorcycle. For each option, the legal guardian will be asked the time of travelling. All these questions are reliable and valid and have been used in several studies [65–67]. Questionnaires are important to assess physical activity because they help to understand the context in which it occurs. However, the questionnaires do not allow to determine the intensity of the activity practised regularly. Besides, children also have difficulty reporting the intensity of activities accurately. Therefore, accelerometers are important to understand the levels of intensity of physical activity practised. To determine the physical activity intensity, children will wear an accelerometer for at least two weekdays and one weekend.

Child sedentary behaviours (e.g. time spent watching television, playing console, reading, or listening to music) [68] and sleep behaviours (number of hours of sleep per day at the weekday and the weekend) will be reported by a legal guardian. These questions are reliable and valid and have already been used in the Portuguese context using national representative samples [66, 69] …

4) Health-Related Quality of Life

The child self-report and parent-report versions of the KIDSCREEN-10 will be applied [70, 71]. Based on the definition of quality of life as a multidimensional construct covering five dimensions (physical, emotional, mental, social, and behavioural components of well-being and functioning), contains 10 items. Each item is answered on a 5-point response scale. Higher values indicate a better health-related quality of life. The KIDSCREEN-10 instrument, version for children and adolescents and version for parents, was adapted and validated for the Portuguese language [71].

5) Social and Emotional Skills

An instrument granted by OECD - Study on Social and Emotional Skills (SSES) - will be applied to evaluate self-regulation, resilience, and adaptability. Children’s legal guardians and children will answer the 32-item (e.g. “the child is not easily annoyed” and “I am not easily annoyed.” respectively) self-report questionnaire using a 5-item Likert scale. Children’s questionnaires will be appliedby the teacher in the classroom and instructions will be readaloud. Teachers will answer the 12-item questionnaire (e.g. “The child can control his/her actions.”), using a 5-item Likert scale.

6) Process Evaluation

The process evaluation will be aimed at all school community members. It will be applied both in close and open-ended questions to children’s legal guardians, teachers, and school staff. The close-ended questions, on a Likert scale, will include global project-related questions (e.g. “How important is the SGH in children’s education?”) and specific questions related to each intervention axis (e.g. “How important is the healthy snacks policy at school?”). The adequacy of the questionnaires applied will also be evaluated (e.g. “Were the questions accessible to answer?”). The open-ended question will aim to collect suggestions and improvement opportunities. Children will answer a self-reported questionnaire, using a three-choice scale, namely how much they liked participating in the curricula health at the table, how much theylearnt, and how much their eating habits became healthier.

7) Anthropometric Data

Weight, height, and waist circumference will be measured objectively, according to standardised procedures [72] and classified according to World Health Organization [73]. Children will be wearing minimal clothing and no shoes to assess height and weight. Height will be assessed using a portable stadiometer to the nearest 0.1 cm (SECA 213®) in the vertical position, with feet together and the head in the Frankfort plan [72]. Weight will be assessed through a portable calibrated scale (SECA 813®), expressed up to 0.1 kg. Waist circumference will be directly measured on the skin to the nearest 0.1 cm, according to the method of Cameron, with a non-extensible and flexible tape (SECA 201®).

8) Snacks Evaluations

During the morning and afternoon snacks, a qualitative and quantitative evaluation will be assessed. A qualitative evaluation grid, containing the foods mentioned in the Directorate-General for Education guidance for school buffets [54], will be applied by the responsible teacher in a random moment at the beginning and the end of the school year. At the same moments, trained researchers will conduct a quantitative and qualitative detailed evaluation of children’s snacks. This assessment will be carried out by direct observation, in random classrooms of each leveland recorded in specific registration grids. For the quantitative analysis, snacks’ nutritional composition will be calculated using a database created by SGH accordingly to the reference values established in the Portuguese Food Composition Table [74].

9) Basic Motor Competences

The MOBAK protocol [75] assesses eight basic motor qualifications, assigned to two areas of motor competence: object-movement (object-movement throwing, catching, bouncing, and dribbling) and self-movement (self-movement balancing, rolling, jumping, and moving sideways). Each score ranges between zero and two points, up to a maximum of eight points in each area, and a maximum score of 16 points for the overall score. A total motor competence category was created (maximum of 16 points) [76].

There will be two different data collection moments per year, one at the beginning and another at the end of each school year (SY). Table 2 describes the information collected at each moment.

**Table 2:** Information collected at each moment of data collection

Data	Beginning of the school year	End of the school year
Written informed consent	Xa	–
Anthropometric data	Xb	Xb
Basic Motor Competences	Xb	Xb
Sociodemographic data	Xa	–
Health-related data, eating habits and behaviours, movement behaviours	Xa	Xa
Health-related quality of life social and emotional skills	Xa,b,c	Xa,b,c
Snacks evaluations	Xb	Xb
Process evaluation	–	Xa, b, c, d

a: children’s legal guardians, b: children; c: teachers; d: school staff

### Data management and statistical analysis

SGH study data will be centralized by the project executive responsible who will hold the participant individuals’ coded key. All individuals involved in the study will be assigned a single anonymised code that will be used to link the respective data across all the existing databases/tables. Each of the performed surveys will have its own database/table with the participant individuals anonymised code.

Regular reporting will be performed using RStudio Notebooks that collect information from the several available databases/tables. This procedure ensures data monitoring, verification, and cleaning. The iterative process of automatic generation of data reporting identifies errors, originated by OCR transposition of paper questionnaires to digital databases/tables, that propel data correction and revision.

The SGH protocol promotes extensive data collection with baseline data points and follow-up points, and with individuals entering and exiting the study during its expected implementation. Overall, this represents a great challenge for the study data management and analysis. For baseline and specific momentary data, statistical characterisation will be performed using descriptive statistics (absolute and relative frequencies for qualitative variables and mean and standard-deviations or median and IQRs for quantitative variables according to verification normality distribution assumption using the Shapiro-Wilk test). Bivariate relations with the main socioeconomic characteristics will be tested using chi-square tests or Fisher exact test (when both variables will be qualitative); Pearson or Spearman’s correlation coefficients (when both variables will be quantitative); and t-, ANOVA or Mann-Whitney and Kruskal-Wallis tests (when one variable will be quantitative and the other qualitative, whether parametric test use will be met or not and according to the discrepancy of sample size between groups). For the comparison of two moments of the same quantitative variable, the paired-samples t-test or the Wilcoxon signed-rank test will be used. For multivariable analyses, the Generalized Linear Models family will be used (it includes Multiple Linear Regression, Multiple Logistic regression, Poisson Regression, and Negative Binomial regression among other models).

## Discussion

Even though there has been a trend towards a decrease in children’s overweight national prevalence since 2008, child obesity remains a major public health concern [12]. Although Portugal has recognised obesity as a chronic disease since 2004 [77], obesity stigma contributes to its underestimation and non-recognition as a serious disease, despite its huge consequences [78]. This stigma might compromise the effectiveness of obesity interventions [78]. Up-to-date community-based interventions for preventing childhood obesity are lacking [43]. Evidence regarding interventions to prevent childhood obesity indicates that multi-component approaches [36, 37], whole community approaches [43], sustained over time [39, 40] and sustainable models [45] are more likely to succeed. Several guidelines reinforce the crucial role of whole-of-government, whole-of-society, and health-in-all-policies approaches in taking action on childhood obesity [9, 79, 80]. Thus, SGH aims to contribute with relevant research to obtain evidence-based practices, providing significant knowledge for policymakers and health and education professionals’ actions.

SGH is aware of its limitations such as scarce research funding opportunities, response rates lower than expected due to low socioeconomic status, namely legal guardians’ education level, intrinsic motivation of the community in the study. SGH community-based participatory research protocol, with its study design and methodology, will ensure better community engagement, associated with partnership effectiveness [81], the sustainability and future reproduction of good practices, through the empowerment of the school community, preventing the increase of childhood obesity prevalence, and improving children’s social and emotional skills and health-related quality of life.

Overall, SGH will innovate by its consistent monitoring and scientific evaluation to establish a sustainable and effective intervention model to prevent childhood obesity. Quality control and assurance of procedures through training of researchers, data collection protocols, processes of internal data quality verification will be taken. To support further policy actions, an annual report addressed to policymakers will be developed. Also, there will be strategic thinking about all the issues concerning the institutional communication of the intervention, as well as social behaviour change communication. Furthermore, SGH scientific dissemination of findings will be assured through a shared publication policy with SGH consortium.

It is crucial to reinforce public investment in health, namely through the presence of more health professionals in local governments and schools, prioritsing the measurement of the impact of their interventions, through partnerships with academic researchers. In addition to having evidence-based practices, it is also essential to obtain more evidence based on practices. Implementing this study protocol will bring relevant scientific findings regarding different factors related to childhood obesity, allowing the development of better strategies to manage this major health problem.

## Data Availability

Not applicable.

## Abbreviations

HPS: Health promoting school
SGH: Sintra Grows Healthy
